# Using National Inpatient Death Rates as a Benchmark to Identify Hospitals with Inaccurate Cause of Death Reporting — Missouri, 2009–2012

**DOI:** 10.15585/mmwr.mm6601a5

**Published:** 2017-01-13

**Authors:** Jennifer Lloyd, Ehsan Jahanpour, Brian Angell, Craig Ward, Andy Hunter, Cherri Baysinger, George Turabelidze

**Affiliations:** 1 Missouri Department of Health and Senior Services.

Reporting causes of death accurately is essential to public health and hospital-based programs; however, some U.S. studies have identified substantial inaccuracies in cause of death reporting. Using CDC’s national inpatient hospital death rates as a benchmark, the Missouri Department of Health and Senior Services (DHSS) analyzed inpatient death rates reported by hospitals with high inpatient death rates in St. Louis and Kansas City metro areas. Among the selected hospitals with high inpatient death rates, 45.8% of death certificates indicated an underlying cause of death that was inconsistent with CDC’s Guidelines for Death Certificate completion. Selected hospitals with high inpatient death rates were more likely to overreport heart disease and renal disease, and underreport cancer as an underlying cause of death. Based on these findings, the Missouri DHSS initiated a new web-based training module for death certificate completion based on the CDC guidelines in an effort to improve accuracy in cause of death reporting.

Among all nonfederal, noninstitutional, short-stay hospitals or general hospitals in Missouri that each reported ≥20 deaths per year, 32 were purposively selected for the study. All selected hospitals were in the Kansas City metro area (15) or the St. Louis metro area (17). Combined, these hospitals reported half (50.7%) of all deaths in the state. Heart disease, cancer, and renal disease were selected from among the 10 top causes of death in the state, because death certificate–based reported deaths resulting from these conditions were substantially higher in Missouri than in the rest of the United States.

Death certificate data from 2009–2012 were obtained from the Missouri Department of Health and Senior Services (MDHSS) Vital Statistics Bureau. Heart disease deaths were defined as deaths assigned *International Classification of Diseases, 10th Revision* (ICD-10) codes I00–I09, I11, I13, or I20–I51; cancer deaths, as those with codes C00–C97; and renal disease deaths, as those with codes N00–N07, N17–N19, or N25–N27.

For each hospital, the average percentage of reported deaths from heart disease, cancer, and renal disease among persons hospitalized for each condition during the study period was calculated as the number of inpatients reported to have died from a particular cause divided by the total number of hospitalizations of persons with a diagnosis of that disease, multiplied by 100 (*1*). An extreme Studentized deviate test to detect multiple outliers in a univariate, approximately normally distributed data set (two-sided test, α = 0.1) was applied to the calculated inpatient hospital death rates data set. Hospitals with high outlying death rates in any of the three disease categories were selected. The rest of the normalized data set was then tested for normality again with the Shapiro-Wilk test (Figure). After calculating the standard deviation (SD) of the normalized data set, the inpatient death data were plotted around the U.S. benchmark, and a tolerance zone (benchmark ±2 SD) was created. CDC’s estimates of the U.S. 2010 inpatient hospital death rates for cancer, heart disease, and renal disease were used as benchmarks (*1*). Among hospitals with inpatient death rates ≥2 SD above the U.S. benchmark in any disease category, a sample of the hospitals that contributed the most deaths were selected. These hospitals, as well as the hospitals identified as outliers, were included in the analysis (Figure).

**FIGURE:**
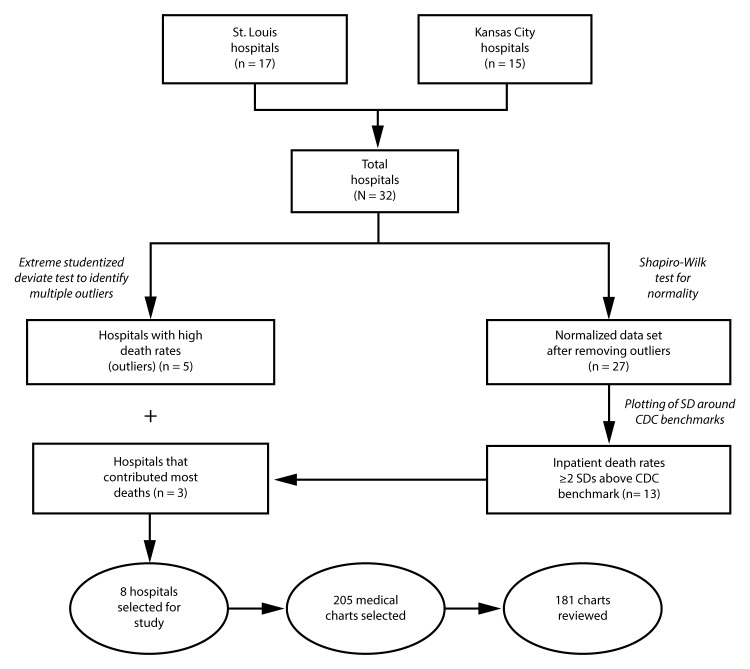
Selection of hospitals for assessment of accuracy of cause-of-death reporting — St. Louis and Kansas City metro areas, Missouri, 2009–2012 **Abbreviation:** SD = standard deviation.

Medical charts for review were randomly selected from a data set that included all death certificates submitted by the hospital during 2009–2012 for the three disease categories. Sample sizes for the chart review were calculated to detect at least a 20% death certificate completion error rate, and ranged from 18 to 33 per hospital. Medical chart reviews were conducted by one physician and one epidemiologist who were trained in death certificate completion according to CDC guidelines (*2*). Death certificates were not available to the reviewers at the time of chart reviews. After a thorough review of medical charts with sufficient data available to determine cause of death, underlying cause of death was determined by consensus between the two reviewers. If the medical chart did not provide sufficient information to reject the cause of death recorded in the chart, the reviewers accepted the diagnosis recorded in the chart. The underlying cause of death determined based on the chart review was subsequently compared with the cause of death recorded on the death certificate. Proportions of deaths from heart disease, cancer, and renal disease as reported on all death certificates were compared with those ascertained through review of the medical chart. Differences were assessed using the McNemar test and a p-value <0.05 was considered statistically significant.

Among the 32 hospitals, five acute care small (<150 beds) hospitals (two in the St. Louis area and three in the Kansas City area) were determined to be outliers with high inpatient death rates (Figure). After setting aside those five hospitals, the resulting normalized data set comprised 27 hospitals: 12 in the Kansas City area and 15 in the St Louis area. Among these, 13 (48%) had inpatient death rates ≥2 SDs above the benchmark for at least one disease category (Table 1). Among these 13 hospitals, three that contributed the most deaths in this group (one in Kansas City and two in St. Louis) were selected by the researchers. These three hospital and the five outlier hospitals constituted the eight study hospitals. A total of 205 medical charts were selected for review at these eight hospitals. Among the 205 selected medical charts, 181 (88%) were reviewed; charts were unavailable or incomplete (e.g., missing notes, no discharge summary, no laboratory results, etc.) for 24 patients.

**TABLE 1:** No. of hospitals that exceeded CDC benchmarks +2 standard deviations* for deaths from heart disease, cancer, and renal disease and all-cause deaths — St. Louis and Kansas City, metro area hospitals, 2009–2012

Reported cause of death	CDC benchmark^†^ (+2 SD)	St. Louis hospitals		Kansas City hospitals	
		No. within tolerance zone	No. outside tolerance zone (no. of outliers)	No. within tolerance zone	No. outside tolerance zone (no. of outliers)
Heart disease	3.5 (5.9)	13	4 (1)	13	2 (2)
Cancer	4.4 (13.7)	11	6 (0)	8	7 (3)
Renal disease	3.1 (7.4)	14	3 (1)	10	5 (1)
All-cause death	2.0 (3.3)	17	0 (0)	15	0 (0)

**Abbreviation:** SD = standard deviation.

* SD is computed after removing outliers.

^†^ Per 100 hospitalizations; benchmark denotes national in-patient death rate

Overall, the cause of death reported on 24%–65% of death certificates submitted by the reviewed hospitals did not agree with the conclusions reached by the chart reviewers: among hospitals studied, heart disease was incorrectly identified as the cause of death on 54.5%–85% of death certificates, renal disease on 0%–44%, and cancer on 0%–9% (Table 2). Three hospitals with high heart disease death rates on the death certificates were more likely to overreport heart disease as an underlying cause of death (odds ratio [OR] = 4.5; 95% confidence interval [CI] = 4.1–90.2). The two hospitals with high renal disease death rates on the death certificates were more likely to overreport renal diseases as an underlying cause of death (p = 0.041). Six hospitals with high cancer death rates on the death certificates were more likely to underreport cancer on the death certificate (OR = 3.7, CI = 1.1–16.4) and overreport heart disease (OR = 9.0, CI = 13.8–25.6) and renal disease (OR = 1.8, CI = 0.6–5.9). As a group, all reviewed hospitals were more likely to overreport heart disease (OR = 6.6, CI = 3.3–14.9) and renal disease (OR = 2.8, CI = 1.04–8.7), but underreport cancer (OR = 4.0, CI = 1.2–17.7) as an underlying cause of death on the death certificate.

**TABLE 2:** Reported underlying cause of death on the death certificate and medical records and percentage of incorrect death certificates, by hospital and disease — St. Louis and Kansas City metro area hospitals, 2009–2012

Hospital	No. medical charts selected	No. medical charts reviewed (%)	% Death certificates with inaccurate cause of death	% Death certificates that inaccurately identified these causes of death		
				Heart disease*	Cancer^†^	Renal disease^§^
A	18	18 (100)	44.0	75.0	0	25.0
B	26	26 (100)	50.0	85.0	0	15.0
C	25	25 (100)	44.0	81.8	9.0	9.0
D	22	22 (100)	41.0	56.0	0	44.0
E	20	20 (100)	65.0	85.0	8.0	0
F	33	24 (73)	45.8	54.5	9.0	27.0
G	31	21 (68)	52.4	54.5	0	18.0
H	30	25 (83)	24.0	83.0	0	0
Mean	NA	NA	45.8	71.9	3.3	17.3

**Abbreviations:** NA = not applicable.

* Defined as deaths assigned *International Classification of Diseases, 10th Revision* (ICD-10) codes I00–I09, I11, I13, or I20–I51.

^† ^Defined as deaths assigned ICD-10 codes C00–C97.

^§^ Defined as deaths assigned ICD-10 codes N00–N07, N17–N19, or N25–N27.

## Discussion

This study revealed substantial overreporting of heart disease and renal disease and underreporting of cancer as underlying causes of death by selected Kansas City and St. Louis area hospitals. Based on review of the medical record by trained reviewers, an average of 45.8% of reviewed death certificates were completed incorrectly. Accuracy of death certificates is of paramount importance, considering that such data are widely used to direct public health projects as well as to fund hospital-based programs and clinical research. However, several studies have demonstrated that death certificates are often completed incorrectly, leading to inaccurate mortality statistics being ascertained from death records (*3*–*6*).

This study was conducted to analyze whether inaccurate death reporting could explain consistently high inpatient death rates for selected conditions at some Missouri hospitals. Because population health risk factors are similar within the geographic region, investigators hypothesized that overreporting of some conditions could, in part, account for increased inpatient death rates associated with certain conditions, and developed an algorithm to identify hospitals with high death rates in both all-cause and selected disease categories. This approach seemed justified considering that all-cause death rates reported by every hospital in this study were comparable to the national rates. Even hospitals with high inpatient cancer death rates underreported cancer as an underlying cause of death at the same time that heart disease was overreported as an underlying cause of death. In those hospitals, the fraction of deaths caused by cancer was consistently and incorrectly identified as caused by heart disease on the death certificate, thereby increasing the heart disease death rate and lowering the cancer-associated death rate. These findings are consistent with previous studies demonstrating that death certificates are often filled out incorrectly (*7*–*10*).

The findings in this study are subject to at least three limitations. First, 12% of medical charts designated for review were unavailable or did not have sufficient information, which might have resulted in sampling bias. Second, the study was based on the assumption that the hospital medical charts provide more accurate representation of the cause of death than the death certificates, although this might not be correct in all cases. Finally, although this study compared cause of death across broad disease categories, the determination of the underlying cause of death is not always straightforward and another reviewer might have reached a different conclusion.

The Missouri DHSS recently implemented a new web-based training module (http://health.mo.gov/training/moevr/certifier/index.html) instructing certifiers in death certificate completion and on-site training for all personnel involved in death records data entry. Monitoring of inpatient death reporting by public health agencies is ongoing to ensure consistent quality of death certificate data considering that these data are widely used to direct health policy locally and nationally.

**Summary**

**What is already known about this topic?**

Inaccurate completion of death certificates affects reliability of mortality statistics routinely used for policy, research, and public health practice.

**What is added by this report?**

Using CDC’s national inpatient death rates data as a benchmark was helpful in identifying hospitals at the local level with high inpatient death rates. Selected hospitals with high inpatient death rates were more likely to overreport heart disease and renal disease, and underreport cancer as an underlying cause of death. A new web-based training module for death certificate completion was initiated in the state for all personnel involved in death records data entry.

**What are the implications for public health practice?**

Because cause of death data are widely used to direct local and national health policy, ongoing monitoring of accuracy of inpatient death reporting by public health agencies is needed to improve reporting.
